# Investigation and systematic review of temporal associations between vaccination and onset of immune-mediated hemolytic anemia or thrombocytopenia in dogs

**DOI:** 10.1093/jvimsj/aalag057

**Published:** 2026-04-08

**Authors:** Victoria Neale, Sophie Broughton, Harriet R Hall, Natalia Caldecott, Amanda Paul, Barbara J Skelly, Barbara Glanemann, James W Swann

**Affiliations:** Anderson Moores Veterinary Specialists, Poles Lane, Hampshire, United Kingdom; Department of Veterinary Medicine, University of Cambridge, Cambridge, United Kingdom; Clinical Science and Services, Royal Veterinary College, Hatfield, United Kingdom; Comparative Biomedical Sciences, Royal Veterinary College, London, United Kingdom; Dick White Referrals, Six Mile Bottom, Cambridgeshire, United Kingdom; Dick White Referrals, Six Mile Bottom, Cambridgeshire, United Kingdom; Anderson Moores Veterinary Specialists, Poles Lane, Hampshire, United Kingdom; Hamilton Specialist Referrals, High Wycombe, United Kingdom; Department of Veterinary Medicine, University of Cambridge, Cambridge, United Kingdom; Clinical Science and Services, Royal Veterinary College, Hatfield, United Kingdom; Clinical Science and Services, Royal Veterinary College, Hatfield, United Kingdom; Columbia Stem Cell Initiative, Columbia University, New York, NY, United States

**Keywords:** adverse drug reaction, autoimmune, hematology, immunization

## Abstract

**Background:**

Vaccination is implicated in development of human immune-mediated diseases, but studies in dogs yielded conflicting results for immune-mediated hemolytic anemia (IMHA) and thrombocytopenia (ITP).

**Hypothesis/Objectives:**

To investigate temporal relationships between onset of IMHA or ITP and vaccination in dogs.

**Animals:**

Client-owned dogs with nonassociative IMHA (*n* = 295) and ITP (*n* = 163) at 4 referral hospitals, alongside control dogs (*n* = 1180 for IMHA, 652 for ITP) presented contemporaneously.

**Methods:**

Multicenter retrospective case–control study, with meta-analysis of published studies. Proportions vaccinated ≤ 30 days before disease onset were compared to controls, along with times since last vaccine.

**Results:**

Proportion of dogs vaccinated ≤ 30 days of disease onset was significantly higher in the IMHA group (35/295, 11.9%) compared with controls (72/1180, 6.1%; *P* = .0015). Dogs with IMHA vaccinated ≤ 30 days of disease onset were more likely to have received a L2 vaccine (*Leptospira* serovars Canicola and Icterohemorrhagiae) in their last dose than those vaccinated > 30 days before onset (*P* = .04). Meta-analysis indicated dogs with IMHA were 2.48 times (95% CI, 1.69-3.63) more likely to have been vaccinated in the past month than controls, but with high heterogeneity across this and 2 other studies. There was no difference in proportion of ITP dogs and controls vaccinated ≤ 30 days of disease onset, and meta-analysis with 1 other study showed no temporal association, though both analyses were likely underpowered.

**Conclusions and clinical importance:**

These results support a possible temporal association between vaccination and onset of IMHA in dogs. Our results do not establish a causal relationship between vaccination and IMHA.

## Introduction

Immune-mediated hemolytic anemia (IMHA) and thrombocytopenia (ITP) are caused by autoimmune destruction of red blood cells or platelets, respectively.^1^ Immunoglobulins coating blood cells trigger their destruction directly through activation of the complement cascade, indirectly by facilitating phagocytosis in the liver and spleen,^2-4^ or through a combination of both mechanisms. Both diseases are diagnosed regularly in small animal referral practice, and both can be considered primary/nonassociative if no underlying cause is detected or can be secondary to various infectious, inflammatory, toxic, or neoplastic conditions.^5-8^

Epidemiological studies and a number of case reports have implicated vaccination as a potential trigger for immune-mediated conditions in human and veterinary medicine,^9-11^ but definitive evidence for causal or temporal associations is currently lacking for IMHA and ITP in dogs.^12^ Evaluating the relationship between vaccination and immune-mediated diseases is important because dog owners report a high level of vaccine hesitancy on behalf of their animals, which is principally driven by concerns about the safety of vaccines.^13^ For IMHA, a temporal relationship was reported between vaccination and development of IMHA in 1996 in a retrospective case–control design^14^: in this study, a higher proportion of dogs with IMHA received a vaccine dose in the month before onset of clinical signs than expected by chance. However, this finding was not replicated in a later retrospective study employing similar methods.^15^ In ITP, some case reports suggest a role for vaccination as a potential trigger for disease in dogs^16^ and humans,^17^^,^^18^ but a retrospective study did not show a temporal association in dogs.^19^ Together, these data show a lack of consensus around temporal associations between vaccination and onset of immune-mediated disease, which could be related to the low case numbers in some studies or variations in vaccine products or population characteristics. Moreover, composition of vaccines has changed considerably over the past 30 years, particularly with the introduction of quadrivalent “L4” (*Leptospira* serovars Canicola, Icterohemorrhagiae, Grippotyphosa, and Bratislava) vaccines for leptospirosis, meaning that results of previous studies might not be relevant to current clinical practice. This lack of certainty regarding a possible association is also reflected in recent consensus statements for diagnosis of both IMHA and ITP.^11^^,^^20^ Collectively, this creates a clinical need to investigate temporal associations between vaccination and onset of immune-mediated diseases in dogs.

The aim of this study was to investigate the temporal relationship between IMHA or ITP and vaccination in a multicenter study. We hypothesized that the proportion of dogs vaccinated within 30 days of onset of clinical signs would be higher in the IMHA/ITP groups compared with matched controls presented for treatment of nonimmune-mediated diseases. Moreover, we conducted a systematic review of published case–control studies investigating this relationship, with the goal of estimating the effect size of any association between recent vaccination and onset of either IMHA or ITP. A second aim was to determine if there were demographic or clinicopathological factors that might be overrepresented among dogs developing IMHA or ITP within 30 days of vaccination compared to dogs that received a vaccine > 30 days before onset of signs.

## Materials and methods

### Study design

A retrospective case–control study was conducted to compare the time of last vaccination and onset of disease in IMHA or ITP cases compared with controls. Ethical approval for the study was granted by the University of Cambridge, Department of Veterinary Medicine Ethical Review Board (CR488).

### Case selection

Medical records for dogs presented to 2 university small animal referral hospitals and 2 private referral hospitals in the United Kingdom (UK) from January 1st, 2008 through December 31st, 2023 were assessed to identify dogs with IMHA or ITP. Search methods varied in each research center owing to differences in practice management software, as outlined in Tables S1 and S2. All cases were managed by clinicians with board certification or eligibility in small animal internal medicine, or residents under their supervision. Inclusion criteria for IMHA cases included all of the following: anemia (PCV < 34%), at least 1 feature of hemolysis (hyperbilirubinemia, bilirubinuria, hemoglobinuria, or erythrocyte ghosts on blood smear), and at least 1 feature of immune-mediated red blood cell destruction (persistent autoagglutination/positive saline agglutination test, positive direct antiglobulin test [DAT], or spherocytosis on blood smear). The inclusion criterion for ITP cases was a platelet count < 40 000/μL at the time of presentation. Evidence of clinical bleeding and coagulation testing were not required for inclusion, but these features were recorded. A CBC and serum biochemistry at the time of presentation were required for all disease cases, along with imaging of the abdomen by ultrasound or computed tomography reviewed by a board-certified imaging specialist or a resident under their supervision. For those dogs with a history of travel outside the UK, serological testing for *Anaplasma* spp., *Ehrlichia* spp., and *Borrelia burgdorferi* and antigen testing for *Dirofilaria immitis* (SNAP 4DX, IDEXX Laboratories) were also required. Clinical history was also required, sufficient to identify the date of last vaccination before onset of disease, along with any medications administered or other illnesses identified within the 3 months before presentation. Cases were excluded if they had suspected underlying disease that could be associated with hemolysis, anemia, thrombocytopenia, or immune-mediated disease, or if the minimum database or clinical records were incomplete. To provide a control sample matched by presentation date, numerical case numbers adjacent to the IMHA/ITP cases were assessed, and 4 control cases were identified for each study case. Exclusion criteria for control cases included any previous diagnosis of immune-mediated disease, lack of definitive diagnosis for current illness that prevented exclusion of immune-mediated disease, species other than dogs, and dogs in which the initial presentation date could not be established. Controls were not matched to cases by age, breed, or sex.

### Data collection

Clinical variables collected for all dogs (cases and controls) included age, sex, breed, travel history, date of presentation, date and type of most recent vaccination before presentation, medications administered, illnesses detected in the 3 months before presentation, and final diagnosis. Breeds were separated into 6 categories based on haplotype sharing (ie, mixed; sight hound and herding; retriever, small terrier, and mastiff; working; toy, spaniel, scent hound; and ancient breeds).^21^ Additional data collected for IMHA and ITP cases included results of CBC (including blood smear examination by a board-certified clinical pathologist), serum biochemistry, presence of spherocytosis, saline agglutination and DAT results, infectious disease testing, and any additional tests performed at the discretion of the attending clinician. For dogs with ITP, a simplified form of the clinical bleeding score DOGiBAT was applied, based on whether signs of bleeding were recorded in the clinical records at 9 different anatomical sites, as reported in the original study.^22^ Since severity of hemorrhage was often not recorded, we applied a score of 1 for each site if bleeding was present and 0 if absent, to give a maximum possible score of 9 for each dog.

### Sample size calculations

Sample size calculations were performed on April 19th, 2021 using online calculators (clincalc.com/stats/samplesize.aspx), with data from a preliminary sample of 54 dogs with IMHA collected at a single center. Data from these dogs were subsequently used in the main analysis. For comparison of proportion of dogs vaccinated in the previous 30 days, we estimated a prevalence of 8% for dogs with IMHA, 4% for controls (representing a 50% reduction in prevalence) with a case: control ratio of 1:4, α 0.05, and power 0.8. This produced a required sample size of 316 cases and 1284 controls. For comparison of the time since last vaccine between cases and controls, we estimated a mean of 216 days for dogs with IMHA from our pilot sample, 238 days for controls (representing a 10% reduction) with case: control ratio of 1:4, α 0.05, and power 0.8. This produced a required sample size of 51 cases and 204 controls. The same samples size calculations were also utilized for cases with ITP.

### Statistical analysis

All statistical analyses were conducted using R (version 4.3.1) and Prism (version 10.4.1, GraphPad software). Shapiro–Wilks tests were used to assess for normality of continuous variables, with normally distributed variables presented as mean and SD, whereas non-normally distributed variables are presented as median and IQR. Age was compared between cases and controls using the Mann–Whitney *U* test. Sex and breed differences were assessed between cases and controls by calculation of odds ratios followed by computation of *P* values by normal approximation. *P* values were corrected for multiple comparisons using the Bonferroni method. Fisher’s exact test was used to compare proportions of dogs in case and matched control groups that were vaccinated within 30 days of onset of clinical signs. Mann–Whitney *U* tests were used to compare times since last vaccination in ITP/IMHA cases and their controls. Dogs with IMHA or ITP were subsequently divided into those presenting within 30 days of vaccination and those presented > 30 days after last vaccination, and binary logistic regression models were constructed to determine whether demographic (age, breed, and sex), vaccination (vaccine product), or clinicopathological variables (PCV; neutrophil, monocyte, and lymphocyte counts; serum bilirubin, albumin, globulin, creatinine, and blood urea nitrogen concentrations; and serum alanine aminotransferase activity) were significant predictors for these groups. Logistic regression models were created with the *glm* function in the R package *stats* (version 3.6.2). For this analysis, breed groups were defined by haplotype sharing, as described above, and entered as a categorical variable with mixed breeds as a reference level. Vaccination data were also coded to indicate whether the last vaccine dose contained antigens for *Leptospira* serovars Canicola and Icterohemorrhagiae (L2); L4; distemper, hepatitis, and parvovirus (DHP); *Bordetella bronchiseptica* (kennel cough, KC); or rabies virus. Data on rabies vaccines were subsequently removed from analysis owing to the low frequency of this vaccine type in the dataset. All vaccination group variables were entered as categorical variables, with absence of the specific vaccine component taken as the reference level. To further evaluate global differences between dogs with IMHA or ITP vaccinated within 30 days or > 30 days of onset of clinical signs, principal component analysis was performed for all clinicopathological variables using the *princomp* function in the R package *stats*, and principal component plots were produced with the *ggplot* function from *ggplot2* (version 3.5.1).

### Systematic review

We conducted a systematic review according to the principles of the PRISMA statement^23^ for published case–control studies that addressed the following PECO question: In dogs presented to veterinary referral centers (P), does recent vaccination (E) compared to no recent vaccination (C) increase the risk of diagnosis of IMHA or ITP (O)? To identify relevant studies, we conducted searches of the Medline/Pubmed and Web of Science (Core Collection) databases between 1980 and 2025 using the search terms shown in Supplementary Information S1. The searches were conducted on August 18th, 2025. Duplicate records were removed and remaining studies were then screened by 1 investigator to exclude those that (1) provided data on species other than dogs, (2) did not contain primary data, (3) were not published in English, (4) described data from < 5 dogs, or (5) related to relapse of immune-mediated diseases. We obtained the full text for the remaining records and further excluded those studies that did not investigate temporal associations between vaccination and onset of IMHA or ITP with a case–control design. Remaining manuscripts were considered for quality assessment, and key results of each study were tabulated by 1 investigator.

### Quality assessment

The quality of included studies was assessed using the Newcastle–Ottawa Scale (NOS) intended for case–control studies,^24^ with each study evaluated independently by 2 different authors. Differences in quality assessment were resolved by consensus.

### Quantitative meta-analysis

For case–control studies with similar designs, quantitative meta-analysis was conducted to compare the results of different studies, with odds ratios calculated to compare the difference in proportion of dogs vaccinated in the 30-42 days before onset of clinical signs. Studies of IMHA and ITP were evaluated separately. Both common-effect (implementing the Mantel–Haenszel method) and random-effects models (using the DerSimonian and Laird method) were then created, and heterogeneity among studies was assessed with the *I*^2^ statistic.^25^ Meta-analysis was performed with the *metabin* function in the R package *meta* (version 8.0-2). For all analyses, statistical significance was defined as *P* < .05.

## Results

### IMHA sample characteristics

Initial searches of medical records identified 5173 dogs with possible IMHA across research centers, of which 295 dogs with a definitive diagnosis of IMHA were included, along with 1180 matched controls (Table S1). Demographic features for each group are shown in Table 1, and comparison between groups revealed significant overrepresentation of neutered females and spaniel breeds among IMHA cases, with underrepresentation of intact and neutered males and retriever breeds. A summary of diagnostic tests, clinicopathological test results, and travel details is outlined in Tables S3–S5. Using criteria outlined in the American College of Veterinary Internal Medicine (ACVIM) consensus statement,^11^ 171 dogs (58.0%) had clinical findings that were “diagnostic” for IMHA, and the remaining 124 dogs (42.0%) had findings that were “supportive.” Major reasons for presentation of control dogs included neurological disease (*n* = 264), neoplasia (223), orthopedic disease (169), gastrointestinal disease (138), respiratory disease (73), trauma (49), urogenital disease (40), cardiac disease (38), endocrine diseases (23), or other reasons (163).

**Table 1:** Demographic variables for dogs with IMHA and controls.

		IMHA	Controls	Odds ratio	95% Confidence interval	*P*
***n***		295	1180			
**Age (median with IQR, years)**		7.1 (4.7-9.0)	6.9 (4.1-9.8)			.98
**Sex**	**Neutered female (*n*, %)**	135 (45.8%)	399 (33.8%)	1.65	1.27-2.14	.00058
	**Neutered male (*n*, %)**	105 (35.6%)	512 (43.4%)	0.72	0.55-0.94	.062
	**Intact female (*n*, %)**	34 (11.5%)	85 (7.2%)	1.68	1.10-2.55	.063
	**Intact male (*n*, %)**	21 (7.1%)	184 (15.6%)	0.41	0.26-0.66	.0010
**Breed group**	**Mixed (*n*, %)**	57 (19.3%)	176 (14.9%)	1.36	0.98-1.90	.039
	**Sight hound and herding (*n*, %)**	12 (4.1%)	78 (6.6%)	0.60	0.32-1.12	.64
	**Retriever, small terrier, and mastiff (*n*, %)**	62 (21.0%)	485 (41.1%)	0.38	0.28-0.52	2.7 × 10^−9^
	**Working (*n*, %)**	23 (7.8%)	64 (5.4%)	1.47	0.90-2.42	.074
	**Toy, spaniel, and scent hound (*n*, %)**	136 (46.1%)	347 (29.4%)	2.05	1.58-2.67	3.9 × 10^−7^
	**Ancient (*n*, %)**	5 (1.7%)	30 (2.5%)	0.66	0.25-1.72	1.0

Abbreviation: IMHA = immune-mediated hemolytic anemia.

### IMHA temporal associations with vaccination

**Figure 1:**
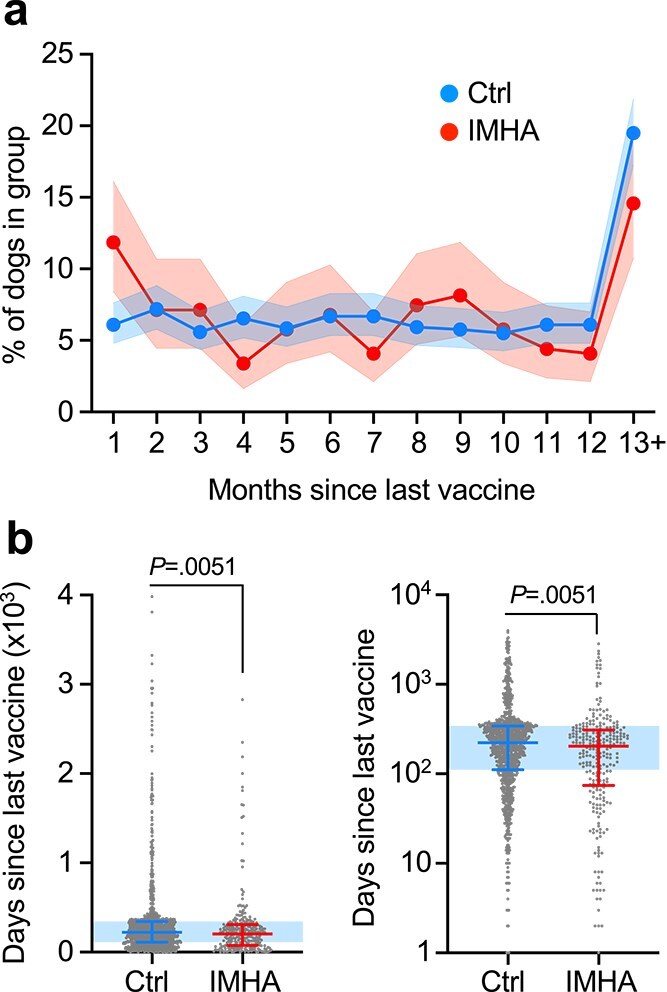
Temporal associations between vaccination and onset of IMHA. (a) Line plot showing proportion of dogs vaccinated at indicated times before diagnosis of IMHA or before presentation for treatment (Ctrl). Shaded areas represent 95% CIs for proportions. (b) Box and whisker plots showing time since last vaccine in IMHA and Ctrl dogs, shown with linear (left) or log (right) scale. Points represent individual dogs with median and interquartile range. Shaded area shows interquartile range for Ctrl dogs. *P* value from Mann–Whitney *U* test. Abbreviations: Ctrl = control; IMHA = immune-mediated hemolytic anemia.

**Figure 2:**
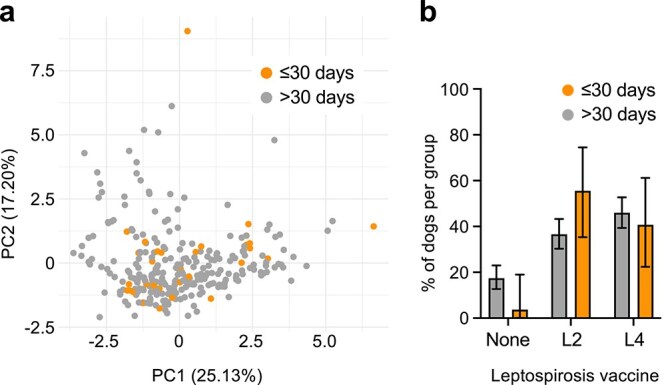
Difference between IMHA dogs according to vaccination timing. (a) Principal component plot generated from clinicopathological data in dogs with IMHA that received their last vaccine dose within 30 days of or > 30 days before onset of clinical signs. Axes show proportion of total variance explained by PCs. (b) Bar plot showing proportion of dogs in each group that received indicated *Leptospira* vaccine types in their last vaccine dose. Bars show proportion within group with 95% CIs. Abbreviations: IMHA = immune-mediated hemolytic anemia; PCs = principal components.

A significantly higher proportion of IMHA cases received a vaccine within 30 days of onset of clinical signs compared to matched controls (IMHA 35/295, 11.9%; controls 72/1180, 6.1%; *P* = .0015) (Figure 1a). Of the dogs presenting within 30 days of vaccination, 15 received a vaccine dose containing L2, 11 L4, 10 DHP, 4 KC, and for 8 dogs, the content of the last vaccine was not known. The most common combination was DHP with L2 (*n* = 7), and a complete summary is outlined in Table S6. The time since most recent vaccination was also significantly shorter for IMHA cases (mean 276 days, median 203 days, IQR: 74-308) compared with matched controls (mean 335 days, median 223 days, IQR: 111-342, *U* = 131 132, *P* = .0051) (Figure 1b). Among IMHA cases, principal component analysis using clinicopathological variables did not reveal any distinct clustering of dogs vaccinated within 30 days or > 30 days before onset of clinical signs (Figure 2a). However, IMHA dogs vaccinated within 30 days of onset of clinical signs were more likely to have received a L2 vaccine compared to dogs vaccinated > 30 days before disease onset (*P* = .04) (Figure 2b; Table 2). There were no other differences in demographic or clinicopathological variables between groups (Tables 3 and 4).

**Table 2:** Comparison of vaccine data between dogs with IMHA that were vaccinated within the previous 30 days and dogs with IMHA vaccinated more than 30 days previously (*n* = 250 dogs, Akaike information criterion: 174.99).

Variable		Estimate	Standard error	*P*
**Leptospirosis**	Not included	Ref		
	L2	2.22	1.07	.04
	L4	1.61	1.07	.13
**DHP**	Not included	Ref		
	Included	−0.39	0.44	.37
**Kennel cough**	Not included	Ref		
	Included	−0.45	0.53	.39

Abbreviations: DHP = distemper, hepatitis, and parvovirus; IMHA = immune-mediated hemolytic anemia; L2 = *Leptospira* serovars Canicola and Icterohemorrhagiae; L4 = *Leptospira* serovars Canicola, Icterohemorrhagiae, Grippotyphosa, and Bratislava.

**Table 3:** Comparison of demographic variables between dogs with IMHA that were vaccinated within the previous 30 days and dogs with IMHA vaccinated more than 30 days previously (*n* = 294 dogs, Akaike information criterion: 228.33).

Variable		Estimate	Standard error	*P*
**Age**		0.07	0.06	.29
**Breed**	Mixed	Ref		
	Sight hound and herding	−0.15	1.15	.89
	Retrievers, small terriers, and mastiffs	0.50	0.60	.40
	Working	0.77	0.74	.29
	Toy, spaniel, and scent hounds	0.20	0.55	.71
	Ancient	1.09	1.25	.38
**Sex**	Female intact	Ref		
	Female neutered	−0.49	0.55	.38
	Male intact	−1.37	1.13	.22
	Male neutered	−0.92	0.59	.12

Abbreviation: IMHA, immune-mediated hemolytic anemia.

**Table 4:** Comparison of clinical pathology variables between dogs with IMHA that were vaccinated within the previous 30 days and dogs with IMHA vaccinated more than 30 days previously (*n* = 268 dogs; Akaike information criterion: 207.09).

Variable		Estimate	Standard error	*P*
**In-saline agglutination**	Absent	Ref		
	Present	1.32	1.06	.21
**Spherocytosis**	Absent	Ref		
	Present	−0.12	0.60	.84
**Neutrophil count**		−0.0067	0.024	.78
**Monocyte count**		−0.049	0.12	.67
**Lymphocyte count**		0.10	0.062	.098
**Serum albumin**		0.047	0.042	.27
**Serum globulin**		0.011	0.026	.68
**Serum total bilirubin**		0.0017	0.0014	.22
**Blood urea nitrogen**		−0.074	0.061	.22
**Serum creatinine**		−0.0040	0.010	.69
**Serum ALT activity**		−0.00020	0.00065	.76
**Packed cell volume**		−0.010	0.036	.78

Abbreviations: ALT = alanine aminotransferase; IMHA = immune-mediated hemolytic anemia.

### ITP sample characteristics

Initial searches of medical records identified 2120 dogs with possible ITP across research centers, of which 163 dogs with a diagnosis of ITP were included, along with 652 matched controls (Table S2). Group demographics are displayed in Table 5, with significant overrepresentation of neutered females and spaniel breeds among ITP cases, whereas intact males and retriever breeds were more frequent among controls. A summary of diagnostic testing, clinicopathological data, and travel information is shown in Tables S7–S9. Although not required for inclusion in this study, coagulation testing was undertaken in 71/163 (43.6%) cases, and serological infectious disease screening was performed in 139/163 (85.3%) cases, even though only 11 dogs (6.7%) had a history of travel outside the UK. In the 71 cases in which coagulation testing was performed, all fulfilled the criteria for “diagnostic” for nonassociative ITP, as defined in the ACVIM consensus statement,^20^ but the remaining cases could not be classified. Upon presentation, 135/163 dogs (82.8%) had evidence of bleeding, and a further 13/163 dogs (8.0%) did not have sufficient information available to establish if clinical bleeding was present, leaving 15/163 dogs (9.2%) with no evidence of clinical bleeding at diagnosis. For those dogs with available information (*n* = 150), the median simplified DOGiBAT score was 2 (IQR: 1-3). The frequency of hemorrhage observed at each anatomical location is shown in Table S10. Reasons for presentation of control dogs included neurological disease (*n* = 139), neoplasia (118), orthopedic disease (106), gastrointestinal disease (60), respiratory disease (46), trauma (25), cardiac disease (25), urogenital disease (16), endocrine (11), and other (106).

**Table 5:** Demographic variables for dogs with ITP and controls.

		ITP	Matched controls	Odds ratio	95% Confidence interval	*P*
***n***		163	652			
**Age (median with IQR, years)**		7.0 (0.5-14.0)	6.5 (0.5-17.0)			.21
**Sex**	**Neutered female (*n*, %)**	74 (45.4%)	208 (31.9%)	1.77	1.25-2.52	.005
	**Neutered male (*n*, %)**	57 (35.0%)	292 (44.8%)	0.66	0.46-0.95	.096
	**Intact female (*n*, %)**	15 (9.2%)	44 (6.7%)	1.40	0.76-2.59	1.00
	**Intact male (*n*, %)**	17 (10.4%)	108 (16.6%)	0.59	0.34-1.01	.0052
**Breed group**	**Mixed (*n*, %)**	29 (17.8%)	121 (18.5%)	0.95	0.61-1.49	1.00
	**Sight hound and herding (*n*, %)**	8 (4.9%)	44 (6.7%)	0.71	0.33-1.55	1.00
	**Retriever, small terrier, and mastiff (*n*, %)**	37 (22.7%)	288 (44.2%)	0.37	0.25-0.55	6.23 × 10^−6^
	**Working (*n*, %)**	6 (3.7%)	24 (3.7%)	1.00	0.40-2.49	1.00
	**Toy, spaniel, and scent hound (*n*, %)**	78 (47.9%)	160 (24.5%)	2.82	1.98-4.03	6.32 × 10^−8^
	**Ancient (*n*, %)**	5 (3.1%)	15 (2.3%)	1.34	0.48-3.75	1.00

Abbreviation: ITP = immune-mediated thrombocytopenia.

### ITP temporal associations with vaccination

**Figure 3:**
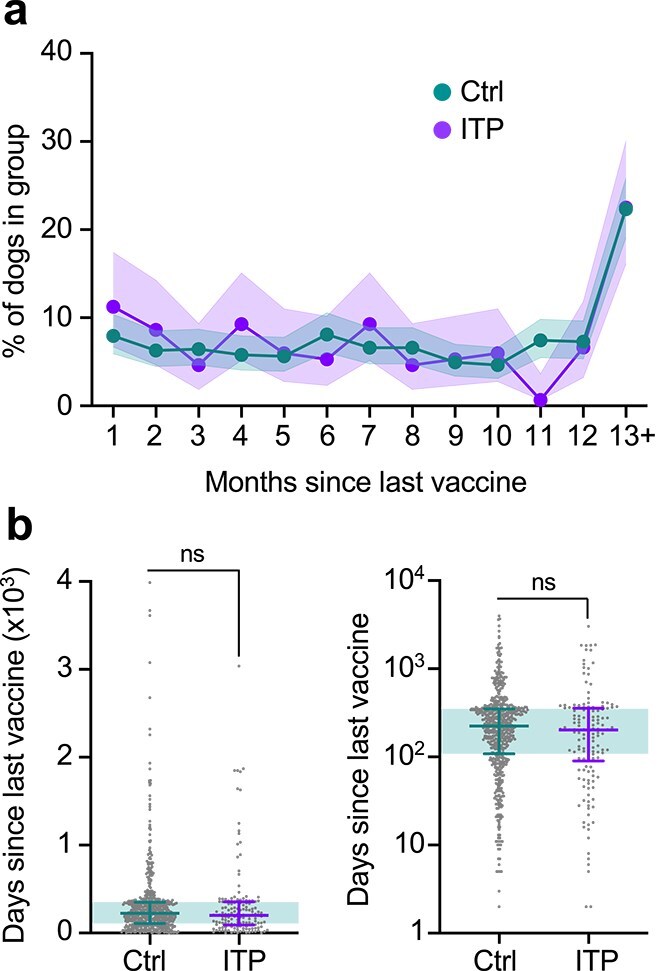
Temporal associations between vaccination and onset of immune thrombocytopenia. (a) Line plot showing proportion of dogs vaccinated at indicated times before diagnosis of ITP or before presentation for treatment (Ctrl). Shaded areas represent 95% CIs for proportions. (b) Box and whisker plots showing time since last vaccine in IMHA and Ctrl dogs, shown with linear (left) or log (right) scale. Points represent individual dogs and median and interquartile ranges. Shaded area shows interquartile range for Ctrl dogs. Mann–Whitney *U* test. Abbreviations: Ctrl = control; ITP = immune-mediated thrombocytopenia; ns = nonsignificant.

**Figure 4:**
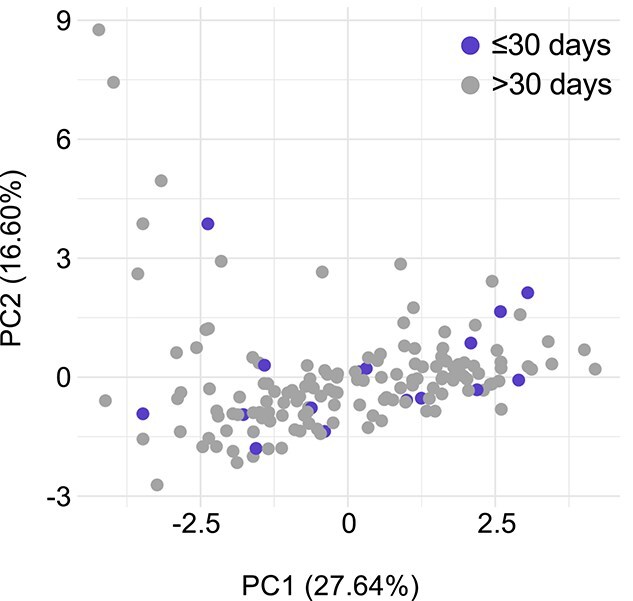
Difference between ITP dogs according to vaccination timing. Principal component plot generated from clinicopathological data in dogs with ITP that received their last vaccine dose within 30 days of or > 30 days before onset of clinical signs. Axes show proportion of total variance explained by PCs. Abbreviations: ITP = immune-mediated thrombocytopenia; PCs = principal components.

There was no significant difference in the proportion of ITP dogs that were vaccinated within 30 days of onset of clinical signs (17/163, 10.4%) compared with their matched controls (49/652, 7.5%, *P* = .26) (Figure 3a), though the number of dogs included in the study did not meet the threshold required to make this calculation with power of 0.8. Conversely, the comparison of time since last vaccination was adequately powered, and we observed no difference when comparing ITP cases (mean 315 days, median 202 days, IQR: 93-351) and controls (mean 322 days, median 224 days, IQR: 109-349, *U* = 42 255, *P* = .15) (Figure 3b). There were also no differences in demographic, clinicopathological, or vaccination parameters between dogs with ITP vaccinated in the past 30 days compared to those vaccinated more than 30 days before onset of clinical signs, nor did these groups cluster separately in principal component analysis (Figure 4; Tables 6–8).

**Table 6:** Comparison of clinical pathology variables between dogs with ITP that were vaccinated within the previous 30 days and dogs with ITP vaccinated more than 30 days previously (*n* = 156 dogs; Akaike information criterion: 119.02).

Variable	Estimate	Standard error	*P*
**Platelet count**	0.03	0.03	.24
**Neutrophil count**	−0.007	0.05	.87
**Monocyte count**	−0.30	0.35	.39
**Lymphocyte count**	0.05	0.15	.76
**Serum albumin**	0.08	0.06	.21
**Serum globulin**	0.01	0.05	.77
**Serum total bilirubin**	−0.001	0.009	.89
**Blood urea nitrogen**	−0.04	0.09	.89
**Serum creatinine**	0.02	0.01	.89
**Serum ALT activity**	−0.0001	0.0003	.71
**Packed cell volume**	−0.06	0.03	.08

Abbreviations: ALT = alanine aminotransferase; ITP = immune-mediated thrombocytopenia.

**Table 7:** Comparison of demographic variables between dogs with ITP that were vaccinated within the previous 30 days and dogs with ITP vaccinated more than 30 days previously (*n* = 163 dogs, Akaike information criterion: 119.05).

Variable		Estimate	Standard error	*P*
**Age**		−0.03	0.09	.78
**Breed**	Mixed	Ref		
	Sight hound and herding	0.18	1.30	.89
	Retrievers, small terriers, and mastiffs	−0.88	0.99	.37
	Working	1.09	1.10	.32
	Toy, spaniel, and scent hounds	−0.07	0.73	.92
	Ancient	−16.69	2869.84	1.00
**Sex**	Female intact	Ref		
	Female neutered	16.87	1667.73	.99
	Male intact	16.66	1667.74	.99
	Male neutered	16.03	1667.74	.99

**Table 8:** Comparison of vaccine data between dogs with ITP that were vaccinated within the previous 30 days and dogs with ITP vaccinated more than 30 days previously (*n* = 143 dogs, Akaike information criterion: 107.80).

Variable		Estimate	Standard error	*P*
**Leptospirosis**	Not included	Ref		
	L2	0.25	1.17	.83
	L4	0.89	1.11	.45
**DHP**	Not included	Ref		
	Included	0.59	0.61	.32
**Kennel cough**	Not included	Ref		
	Included	0.23	0.71	.75

Abbreviations: DHP = distemper, hepatitis, and parvovirus; L2 = *Leptospira* serovars Canicola and Icterohemorrhagiae; L4 = *Leptospira* serovars Canicola, Icterohemorrhagiae, Grippotyphosa, and Bratislava.

### Systematic review of vaccination case–control studies

**Figure 5:**
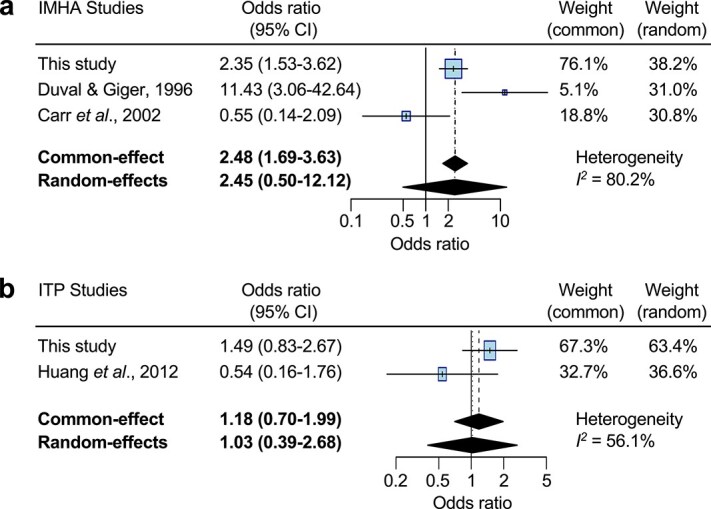
Meta-analysis of vaccination studies. (a and b) Forest plot and parameters for meta-analysis of studies of temporal associations between vaccination and IMHA (a) or ITP (b). Abbreviations: IMHA = immune-mediated hemolytic anemia; ITP = immune-mediated thrombocytopenia.

Initial literature searches identified 351 articles relating to vaccination and IMHA in dogs, and 308 for vaccination and ITP in dogs. After removing duplicates and irrelevant references, 2 articles were included for IMHA, and 1 for ITP, alongside the current study. Reasons for exclusion of the remaining studies are shown in Figures S1 and S2, and the results of included case–control studies are summarized in Table 9. Quality assessment revealed that all studies had low levels of possible bias across most domains of the NOS, especially for case selection and methods of assessing vaccine exposure, with higher possible bias for selection of controls, which were exclusively derived from hospital populations, and for reporting of cases/controls in which vaccine exposure could not be determined (Table 10). For quantitative meta-analysis, consideration of 3 case–control studies for dogs with IMHA^14^^,^^15^ revealed an overall odds ratio of 2.48 (common-effect, 95% CI, 1.69-3.63; *P* < .0001) and 2.45 (random-effects, 95% CI, 0.50-12.12; *P* = .27) (Figure 5a), indicating that dogs with IMHA were ~ 2.4 times more likely to have received a vaccine within 30 days of onset of clinical signs than control dogs across studies. Given the high level of heterogeneity among studies (*I*^2^ = 80.2%), the random-effects model is likely to provide a more accurate estimate of effect size, but the 95% CI for this model also includes the null hypothesis of no difference between groups. For ITP, meta-analysis of 2 studies^19^ showed combined odds ratios of 1.18 (common-effect, 95% CI, 0.70-1.99; *P* = .54) and 1.03 (random-effects, 95% CI, 0.39-2.68; *P* = .96) (Figure 5b), indicating that dogs with ITP were no more likely than control dogs to have been vaccinated within 30-42 days of onset of clinical signs. The PRISMA checklist for the systematic review is provided in Supplementary Information S2.

**Table 9:** Summary of results for published studies included in systematic review.

Citation	Case inclusion criteria	Case exclusion criteria	Control inclusion criteria	Control exclusion criteria	Case dogs (*n*)	Control dogs (*n*)	Time period	Cases vaccinated (%)	Controls vaccinated (%)	*P* value (Fisher’s exact test)
***IMHA***										
**Duval and Giger, 1996**	Anemia, and hemolysis, and positive DAT or saline agglutination after washing	Drug or toxin, or infectious disease, or neoplasia, or vaccine history not available	Complete vaccine records, and no history of anemia, IMHA, or other immune-mediated disease	None stated	58	70	1 month	15/58 (25.9%)	3/70 (4.3%)^a^	.0006
**Carr et al, 2002**	PCV < 34%, and 1 of following: persistent autoagglutination, positive DAT, regenerative anemia with spherocytosis	Any underlying cause, including neoplasia, incomplete records/data, and infections	None stated	None stated	52	33	1 month	5/52 (9.6%)	5/33 (15.2%)	.5005
***ITP***										
**Huang et al, 2012**	Plt < 40 000/μL, and clinical signs of hemorrhage, and PT/PTT no more than 25% above RI, and negative antibody titer for *Ehrlichia canis.* Complete vaccine history	Any evidence for underlying cause, after thoracic and abdominal imaging, and exposure to drugs associated with ITP in previous 42 days	No history of immune-mediated disease, and complete vaccine records, and normal platelet count	None stated	48	96	42 days	4/48 (8.3%)	13/96 (13.5%)	.4242

a This is derived from Figure 1, which shows that 5% of control dogs were vaccinated in the month before presentation, but that 10/70 control dogs were excluded.

Abbreviations: DAT = direct antiglobulin test; IMHA = immune-mediated hemolytic anemia; ITP = immune-mediated thrombocytopenia; Plt = platelet count; PT = prothrombin time; PTT = partial thromboplastin time; RI = reference interval.

**Table 10:** Results of quality assessment for studies included in systematic review.

	Newcastle–Ottawa Scale domain								
Study	Selection: case definition	Selection: represent-ativeness	Selection: control selection	Selection: control definition	Compara-bility: timing of presentation	Compara-bility: age, sex, and breed matching	Exposure: ascertainment	Exposure: same methods for cases and controls	Exposure: nonresponse rate
***IMHA***									
**This study**	^a^	^a^	b) Hospital controls	^a^	^a^	b) No matching	^a^	^a^	d) Not recorded
**Duval and Giger, 1996**	^a^	^a^	b) Hospital controls	^a^	^a^	b) No matching	^a^	^a^	d) Not reported for controls
**Carr et al., 2002**	^a^	^a^	b) Hospital controls	b) No description of source	^a^	b) No matching	^a^	^a^	b) Nonrespondents described
***ITP***									
**This study**	^a^	^a^	b) Hospital controls	^a^	^a^	b) No matching	^a^	^a^	d) Not recorded
**Huang et al., 2012**	^a^	^a^	b) Hospital controls	^a^	^a^	^a^(Age-matched)	^a^	^a^	d) Not reported

a Indicates lowest risk of bias in indicated domain.

Abbreviations: IMHA = immune-mediated hemolytic anemia; ITP = immune-mediated thrombocytopenia.

## Discussion

Vaccines are implicated as possible triggers for immune-mediated diseases, but available evidence for dogs with IMHA and ITP was contradictory and produced before the development of some current vaccine formulations. Here, we find in a large, multicenter study that dogs with IMHA were more likely to have been vaccinated in the 30 days before onset of clinical signs than controls, but we did not observe any association between vaccination and onset of signs in dogs with ITP when comparing time since last vaccine dose. Dogs with IMHA that received a vaccine in the 30 days before onset of clinical signs were more likely to have received a L2 vaccine product in their last vaccine dose compared to dogs with IMHA that received their last vaccine dose > 30 days before onset of clinical signs. Our results do not establish a causal relationship between vaccination and immune-mediated diseases.

Our findings in this study support those of a previous report,^14^ which described a similar temporal relationship between vaccination and onset of clinical signs in dogs with IMHA. Conversely, a later study did not observe the same relationship, but we speculate that this might be attributable to the low number of control cases available for comparison in that study, or to differences in sample characteristics or vaccine products. Notably, our work included considerably more dogs than previous studies and, for dogs with IMHA, we approximated the number of cases needed to conduct all of our planned statistical analyses with power of 80%. Moreover, by combining all studies in a meta-analysis, our models suggest that dogs with IMHA are overall ~ 2.4 times more likely to have been vaccinated in the 30 days before disease onset than controls presented for nonimmune-mediated diseases, but the high level of heterogeneity among studies suggests that additional independent investigations are needed to provide more accurate estimates of population risk. For ITP, our results resemble those of a previous study that reported no temporal relationship between vaccination and onset of thrombocytopenia,^19^ and our meta-analysis suggests no overall association. While the comparison of time between vaccination and development of ITP was adequately powered and did not show a significant association, the comparison of the proportion of cases presenting within 30 days was insufficiently powered for dogs with ITP. Larger studies of dogs with ITP are therefore required to exclude the possibility of type II error for temporal associations with vaccination, and larger sample sizes could also reveal smaller differences in time since last vaccine than the 10% difference for which our study was adequately powered.

In humans, a variety of vaccines including measles, mumps, and rubella; hepatitis B; tick-borne encephalitis; influenza; and severe acute respiratory syndrome coronavirus 2 (SARS-CoV-2) vaccines are associated with development of ITP and IMHA, or IMHA.^17^^,^^18^^,^^26-31^ One explanation for these temporal associations, including the link we observe in dogs with IMHA, is that vaccination could erode immune tolerance, increasing the likelihood of immune responses directed at self-antigens. Possible mechanisms for such an effect include molecular mimicry between vaccine and self-antigens, nonspecific activation of lymphocytes by vaccine adjuvants, and epitope spreading in immune responses.^32^ To understand whether vaccination might be affecting immune regulation in dogs, further investigations are warranted to characterize the immunophenotype and antigen receptor repertoires of those IMHA/ITP cases diagnosed shortly after exposure. Since measurement of antiplatelet antibodies is not a common procedure in dogs, it is possible that some presumptive diagnoses of ITP after vaccination are not driven by immune-mediated platelet destruction. Moreover, while a recent experimental study documented an increase in antiplatelet antibodies in a dog after combined distemper, adenovirus-2, parainfluenza, and parvovirus vaccination, this was actually associated with an increase in platelet count and no evidence of clinical bleeding,^33^ suggesting that vaccine-induced antiplatelet immune responses might not reach a threshold to cause ITP. Indeed, recent data from people receiving adenoviral vaccines for SARS-CoV-2 suggest development of ITP is dependent on specific somatic hypermutation events, which result in production of antibodies specific for platelet factor 4.^34^

Comparison of dogs with IMHA that received a vaccine dose within 30 days or > 30 days before onset of clinical signs identified a significantly higher rate of L2 vaccines among the former group. However, this conclusion is complicated by the fact that many dogs received other vaccines at the same time, meaning that associations might represent more complex interactions among vaccine constituents. We were interested to note a possible association with L2 vaccines when there was considerable anecdotal concern about a possible higher rate of adverse effects upon the introduction of L4 vaccines, for which we did not observe any association. Indeed, the UK authority that monitors adverse responses to vaccines considers any adverse effects associated with both L2 and L4 products to be “very rare,” with rates less than 1 per 10 000 exposed dogs.^35^ Rabies vaccination has also been associated with immune-mediated reactions, particularly cutaneous vasculitis,^36^ but the uptake of rabies vaccination is generally low in the UK after eradication of the virus in 1922, precluding analysis in our study. Possible temporal associations between rabies vaccination and immune-mediated diseases might be of much greater relevance in territories where this practice is routine, as in the United States. Regardless of vaccine product, our study highlights the critical importance of reporting suspected adverse drug and vaccine responses to competent authorities so that possible associations can be monitored over time.

Akin to IMHA and ITP, studies of other immune-mediated diseases in dogs have produced mixed results, with a recent finding of temporal association with vaccination for meningoencephalitis of unknown etiology,^37^ but no association found for steroid-responsive meningitis arteritis.^38^ Moreover, 2 recent studies did not find any association between timing of last vaccination and relapse in dogs with immune-mediated diseases including IMHA, though both included only small numbers of cases.^10^^,^^39^ These findings raise the notion that vaccines could influence specific types of autoimmune response directed at particular cell types, rather than affecting global immune tolerance to diverse tissues.

Our study has several limitations. Although a limit of 30 days has been used traditionally to consider a possible association with vaccination, this is arbitrary and might lead to underestimation of possible vaccine-associated cases. Both the IMHA/ITP and control dogs were recruited exclusively from referral practices, meaning that case and control groups might not be representative of the wider population of dogs. Although all dogs with IMHA met criteria for the “supportive” category of a recent consensus statement, which includes direct evidence of immune-mediated erythrocyte destruction^11^ such as spherocytosis, positive DAT, or in-saline agglutination, the precise methodology for these tests was not recorded, which might have limited the robustness of the diagnosis. Finally, meta-analysis conducted with small numbers of studies could be biased by the largest study or by single studies with small numbers of enrolled cases.

Dogs with ITP were diagnosed after excluding other causes of thrombocytopenia, without any test that detects platelet destruction directly. Cases records were also reviewed in this study to exclude those cases suspected of having other causes of thrombocytopenia, but we did not state explicitly in our data collection process that this should include dogs with any evidence of sequestration, consumption, or decreased platelet production. Nevertheless, since all cases were presented for investigation at specialist centers and because the platelet count was very low in most cases, we consider it unlikely that dogs with these other causes of thrombocytopenia were included in the study. In addition, coagulation testing was not a requirement for definitive diagnosis of ITP in this study, as recommended in a recent ACVIM consensus statement,^20^ meaning that other causes of coagulopathy could not be excluded definitively. For both ITP and IMHA, we required only abdominal imaging to exclude possible underlying conditions because recent studies suggest that thoracic imaging rarely identifies any possible trigger for these diseases,^40-42^ meaning that some cases with associative disorders could have been missed. Similarly, since the prevalence of vector-borne diseases is low in the UK,^43^ infectious disease testing was only required for cases with international travel history, meaning that infectious agents could not be excluded entirely in some cases. Moreover, recent studies have reported rare cases of vector-borne diseases, including *Ehrlichia* spp. and *Babesia* spp., in dogs with no known travel history residing in the UK,^44^^,^^45^ meaning that rare autochthonous infections could not be excluded for those pathogens in untested dogs. The most common method of infectious disease screening for cases that traveled outside the UK was via serological testing for *Anaplasma* spp., *Ehrlichia* spp., and *B burgdorferi* and antigen testing for *D immitis* (SNAP 4DX, IDEXX Laboratories). Given the lack of concurrent molecular or other antigen testing, it is possible that some cases could have been misdiagnosed, but we consider the possible impact of any undiagnosed infectious diseases to be low in this study owing to the small proportion of traveled cases and the rarity of autochthonous infections.

In conclusion, we observed a temporal association between vaccination and development of IMHA in dogs, but not in dogs with ITP when comparing time since last vaccine dose, although this latter finding could have been underpowered. The only variable that distinguished IMHA dogs vaccinated within 30 days from IMHA cases that received their last vaccine > 30 days previously was a higher frequency of L2 products in the last vaccine dose. This work emphasizes the importance of obtaining an accurate vaccine history in dogs with IMHA, as well as accurate reporting of suspected adverse vaccine reactions to competent authorities.

## Supplementary Material

Supplementary_Figures_aalag057

Supplementary_Information_1_aalag057

Supplementary_Information_2_R2_aalag057

Supplementary_Tables_R2_aalag057

## Off-label antimicrobial declaration

The authors declare no off-label use of antimicrobials.

## Institutional animal care and use committee or other approval declaration

Approved by the University of Cambridge Ethical Review Board (CR488).

## Abbreviations

DAT: direct antiglobulin test
DHP: distemper, hepatitis, parvovirus
IMHA: immune-mediated hemolytic anemia
ITP: immune-mediated thrombocytopenia
KC: kennel cough
L2: *Leptospira* serovars Canicola and Icterohemorrhagiae
L4: *Leptospira* serovars Canicola, Icterohemorrhagiae, Grippotyphosa, and Bratislava
SARS-CoV-2: severe acute respiratory syndrome coronavirus 2
