# Supplementary figures and images for: Investigation and systematic review of temporal associations between vaccination and onset of immune-mediated hemolytic anemia or thrombocytopenia in dogs
Source: J Vet Intern Med. 2026 Apr 8;40(2):aalag057. doi: 10.1093/jvimsj/aalag057 (PMC13069898; doi:10.1093/jvimsj/aalag057)

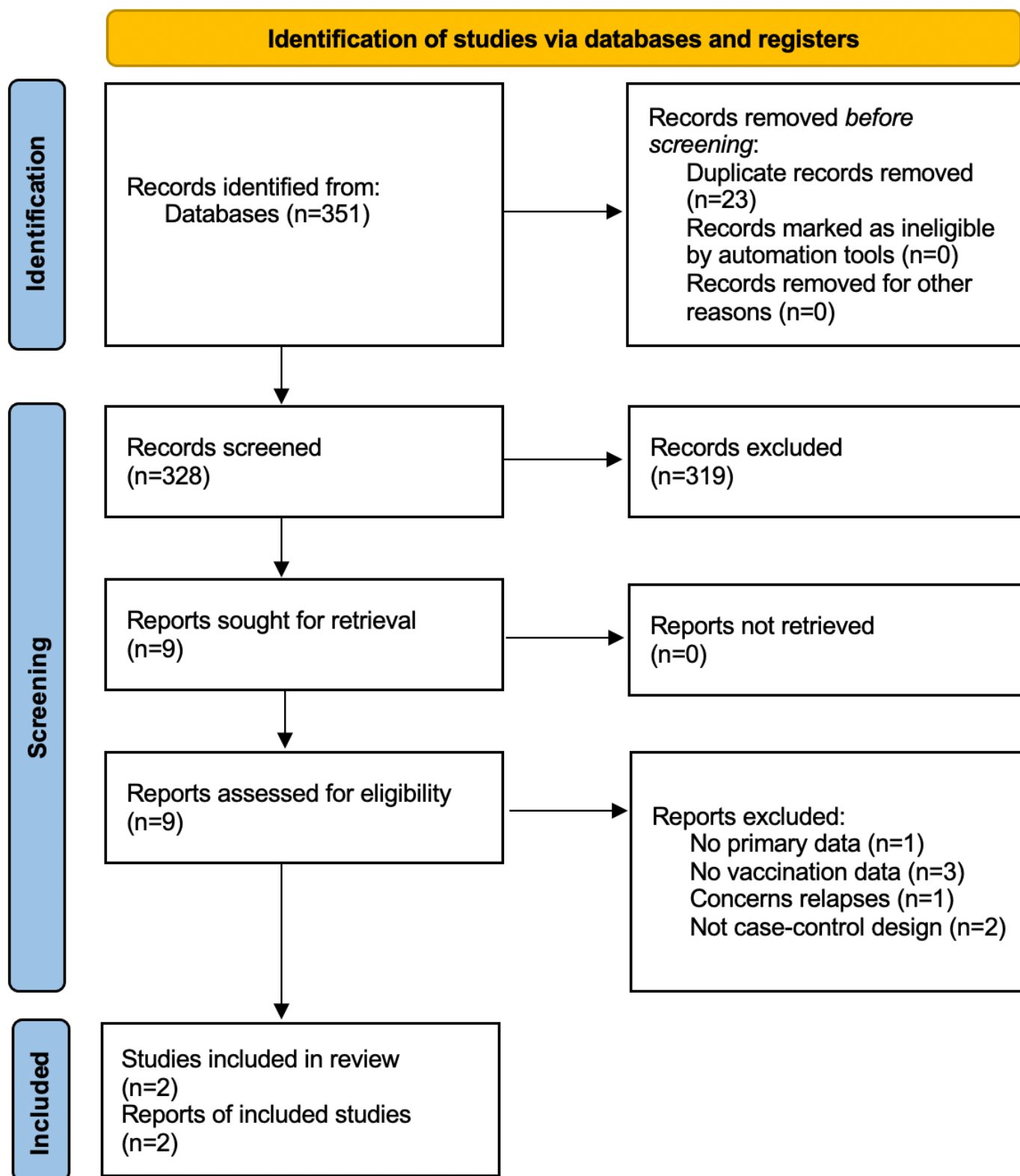

**Supplementary Figure 1**

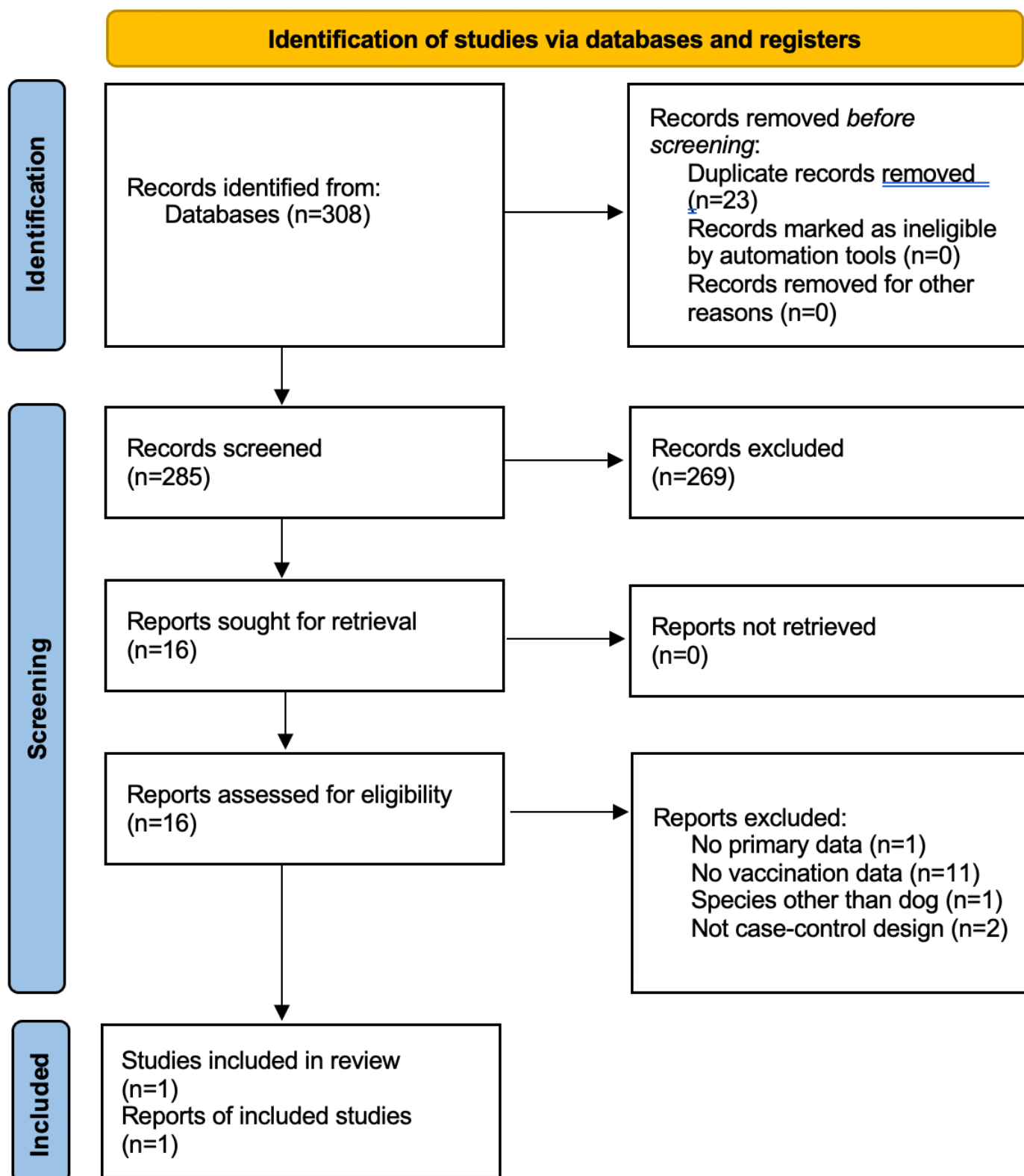

**Supplementary Figure 2**

Supplement: Supplementary_Figures_aalag057 [file supplementary_figures_aalag057.pdf]
