# Supplementary material for: Investigation and systematic review of temporal associations between vaccination and onset of immune-mediated hemolytic anemia or thrombocytopenia in dogs
Source: J Vet Intern Med. 2026 Apr 8;40(2):aalag057. doi: 10.1093/jvimsj/aalag057 (PMC13069898; doi:10.1093/jvimsj/aalag057)
Supplement: Supplementary_Information_1_aalag057 [file supplementary_information_1_aalag057.docx]

**Supplementary Information 1:** Search terms used for systematic review literature search

**IMHA:**

(immune-mediated hemolytic anemia OR immune mediated hemolytic anemia OR immune-mediated haemolytic anaemia OR immune mediated haemolytic anaemia OR imha OR aiha OR autoimmune hemolytic anemia OR autoimmune haemolytic anaemia) AND

(“Dogs”[Mesh] OR dog OR canine OR canis familiaris) AND

(“Vaccination”[Mesh] OR vaccin* OR immunisation OR immunization)

**ITP:**

("Purpura, Thrombocytopenic, Idiopathic"[Mesh] OR ITP OR immune thrombocytopenia OR immune-mediated thrombocytopenia OR IMTP OR IMT) AND

(“Dogs”[Mesh] OR dog OR canine OR canis familiaris) AND

(“Vaccination”[Mesh] OR vaccin* OR immunisation OR immunization)
