# Supplementary material for: Investigation and systematic review of temporal associations between vaccination and onset of immune-mediated hemolytic anemia or thrombocytopenia in dogs
Source: J Vet Intern Med. 2026 Apr 8;40(2):aalag057. doi: 10.1093/jvimsj/aalag057 (PMC13069898; doi:10.1093/jvimsj/aalag057)
Supplement: Supplementary_Tables_R2_aalag057 [file supplementary_tables_r2_aalag057.docx]

**Supplementary Table 1:** IMHA search criteria and excluded cases separated by research center

|  | **Center 1** | **Center 2** | **Center 3** | **Center 4** | **Total** |
| --- | --- | --- | --- | --- | --- |
| **Total raised by search (N)** | 220 | 83 | 2591 | 2279 | 5173 |
| **Cases excluded owing to following reasons (N):**   - **Not canine** - **Not IMHA** - **No imaging** - **Associative disease** - **Insufficient history** | 125 | 35 | 2482 | 2236 | 4873 |
| **Included (N)** | 95 | 48 | 109 | 43 | 295 |

Methods of searching medical records and search criteria:

- Center 1: Practice management software search for “canine AND (IMHA OR immune mediated haemolytic anaemia)”
- Center 2: Case log search for “canine AND (IMHA OR immune mediated haemolytic anaemia)”
- Center 3: Search of clinical pathology records with PCV <35% AND canine
- Center 4: Practice management software search for “canine AND (IMHA OR immune mediated haemolytic anaemia)”. Previous case logs also evaluated for any missing cases.

**Supplementary Table 2:** ITP search criteria and excluded cases separated by research center

|  | **Center 1** | **Center 2** | **Center 3** | **Center 4** | **Total** |
| --- | --- | --- | --- | --- | --- |
| **Total raised by search (N)** | 88 | 70 | 352 | 1610 | 2120 |
| **Cases excluded owing to following criteria (N):**   - **Not canine** - **Not ITP** - **No imaging** - **Associative disease** - **Insufficient history** | 47 | 39 | 296 | 1575 | 1957 |
| **Included (N)** | 41 | 31 | 56 | 35 | 163 |

Methods of searching medical records and search criteria:

- Center 1: Practice management software search for “canine AND (IMTP OR ITP OR immune mediated thrombocytopenia OR immune mediated thrombocytopaenia)”.
- Center 2: Case log search for “canine AND (IMTP OR ITP OR immune mediated thrombocytopenia OR immune mediated thrombocytopaenia)”.
- Center 3: Search of clinical pathology records with automated platelet count < 40,000/μL AND canine
- Center 4: Practice management software search for “canine AND (IMTP OR ITP OR immune mediated thrombocytopenia OR immune mediated thrombocytopaenia)”. Previous case logs also evaluated for any missing cases.

**Supplementary Table 3:** Diagnostic investigations performed in IMHA cases

| **Test Performed** | **Number of cases** |
| --- | --- |
| **Presence of spherocytosis (positive results)** | 288 (252) |
| **Saline agglutination (positive results)** | 287 (263) |
| **Direct antiglobulin test (positive results)** | 103 (78) |
| **Abdominal imaging** | 295 |
| **Thoracic imaging** | 288 |
| **4DX infectious disease serology** | 215 |
| **Cytology and/or histology**  (bone marrow, hepatic, splenic, lymph node CSF, abdominal fluid, pleural fluid and bronchoalveolar lavage fluid and gastrointestinal) | 82 |
| **Urinalysis** | 72 |
| **Blood typing** | 73 |
| **Coagulation testing**  (OSPT/APTT, D dimers, VCM, BMBT) | 46 |
| **Additional infectious disease testing** (leptospirosis serology and PCR, *Ehrlichia* PCR, *Anaplasma* PCR, *Babesia* PCR, *Hepatozoon* PCR, *Bartonella* PCR, parvovirus antigen SNAP test, *Angiostrongylus vasorum* antigen testing (AngioDetect^®^ SNAP test, Baermann), Leishmania serology) | 37 |
| **Additional imaging**  (echocardiography, brain MRI, CT head, spinal radiography) | 18 |
| **Endocrine and metabolic testing**  (TT4/TSH, basal cortisol, plasma metanephrins, ACTH stimulation test, fructosamine urinalysis, cPLI, folate, cobalamin, TLI, ionised calcium, bile acids) | 8 |
| **Fecal analysis** | 5 |
| **Phosphofructokinase genetic testing** | 1 |
| **Serum protein electrophoresis** | 1 |

**Supplementary Table 4:** Clinicopathological variables for IMHA cases.

| **Variable** | **Median (IQR)** |
| --- | --- |
| **PCV (%)** | 16 (12-19) |
| **Red blood cells (x10^6^/μL)** | 1.87 (1.33-2.54) |
| **Hemoglobin (g/dL)** | 5.1 (3.7–6.8) |
| **Neutrophils (x10^3^/μL)** | 15.1 (9.2-22.2) |
| **Band neutrophils (x10^3^/μL)** | 1.04 (0.42-2.57) |
| **Monocytes (x10^3^/μL)** | 1.74 (1.07-3.30) |
| **Lymphocytes (x10^3^/μL)** | 1.67 (1.02-2.85) |
| **Manual Platelet count (x10^3^/μL)** | 185 (85-312) |
| **Automated Platelet count (x10^3^/μL)** | 189 (111-320) |
| **Albumin (g/dL)** | 3.0 (2.7-3.2) |
| **Globulin (g/dL)** | 3.6 (3.2-4.1) |
| **Bilirubin (mg/dL)** | 0.12 (0.06-0.32) |
| **Urea (mg/dL)** | 21.0 (14.8-28.6) |
| **Creatinine (mg/dL)** | 0.63 (0.49-0.83) |
| **Alanine aminotransferase activity (U/L)** | 49.5 (27.0-112.0) |

**Supplementary Table 5:** Travel history for IMHA cases

|  | **IMHA** |
| --- | --- |
| **Total number of cases with travel history (%)** | 22 (7.3) |
| **France** | 5 |
| **Netherlands** | 3 |
| **Portugal** | 2 |
| **Romania** | 2 |
| **Unknown destination** | 2 |
| **Germany, Brazil, Ecuador, Canada, Israel, Singapore, Spain, Greece** | 1 |

**Supplementary Table 6:** Vaccination types for IMHA cases presented within 30 days of vaccination.

| **Vaccination** | **Number of cases** |
| --- | --- |
| **Unknown** | 8 |
| **DHPPi + L2** | 7 |
| **L2** | 6 |
| **L4** | 6 |
| **L4 + KC; DHPPi + L4** | 2 |
| **Pi + L2; KC; L2 + KC; DHPPi + L4 + KC** | 1 |

DHPPi: distemper, hepatitis, parovirus, and parainfluenza viruses; KC: kennel cough; L: leptospirosis; Pi: parainfluenza virus.

**Supplementary Table 7**: Diagnostic investigations performed in ITP cases

| **Test Performed** | **Number of cases** |
| --- | --- |
| ***Angiostrongylus vasorum* testing (Baermann’s, AngioDetect®, PCR)** | 82 |
| **Abdominal imaging** | 163 |
| **Thoracic imaging** | 161 |
| **4DX infectious disease serology** | 139 |
| **Cytology and/or histology**  (bone marrow, hepatic, splenic, lymph node CSF, abdominal fluid, pleural fluid and bronchoalveolar lavage fluid and gastrointestinal) | 10 |
| **Urinalysis** | 23 |
| **Blood typing** | 8 |
| **Coagulation testing**  (OSPT/APTT, D dimers, VCM, BMBT) | 71 |
| **Additional infectious disease testing** (leptospirosis serology and PCR, *Ehrlichia* PCR, *Anaplasma* PCR, *Babesia* PCR, *Hepatozoon* PCR, *Bartonella* PCR, parvovirus antigen SNAP test, *Leishmania* serology) | 10 |
| **Additional imaging**  (echocardiography, brain MRI, CT head, spinal radiography) | 5 |
| **Endocrine and metabolic testing**  (TT4/TSH, basal cortisol, plasma metanephrins, ACTH stimulation test, fructosamine urinalysis, cPLI, folate, cobalamin, TLI, ionised calcium, bile acids) | 4 |
| **Fecal analysis** | 3 |

**Supplementary Table 8:** Clinicopathological results for ITP cases

| **Variable** | **Median (IQR)** |
| --- | --- |
| **Platelets (x10^3^/μL)** | 7 (3-13) |
| **Red blood cells (x10^6^/μL)** | 4.31 (2.64-5.99) |
| **Hemoglobin (g/dL)** | 10.4 (6.4-14.3) |
| **Neutrophils (x10^3^/μL)** | 11.44 (7.26-17.40) |
| **Band neutrophils (x10^3^/μL)** | 0.53 (0-2.14) |
| **Monocytes (x10^3^/μL)** | 1.28 (0.66-2.14) |
| **Lymphocytes (x10^3^/μL)** | 1.71 (1.09-2.69) |
| **Albumin (g/dL)** | 2.7 (2.3-3.1) |
| **Globulin (g/dL)** | 3.0 (2.5-3.4) |
| **Bilirubin (mg/dL)** | 0.25 (0.18-0.38) |
| **BUN (mg/dL)** | 18.2 (12.9-23.0) |
| **Creatinine (mg/dL)** | 0.71 (0.52-0.88) |
| **Alanine aminotransferase activity (U/L)** | 41.5 (26.0-73.5) |

**Supplementary Table 9:** Travel history for ITP cases

|  | **ITP** |
| --- | --- |
| **Total number of cases with travel history (%)** | 11 (6.7) |
| **France** | 4 |
| **Spain** | 3 |
| **Romania** | 2 |
| **Mexico** | 1 |
| **Unknown destination** | 1 |

**Supplementary Table 10**: Clinical bleeding scores and location for n=150 ITP cases that had available medical record information.

| **Anatomical location of bleeding** | **Cases, N (%)** |
| --- | --- |
| Skin | 84 (56.0%) |
| Cather/venepuncture site | 25 (16.7%) |
| Oral mucosa | 96 (64.0%) |
| Intraocular | 23 (15.3%) |
| Epistaxis | 13 (8.7%) |
| Gastrointestinal | 81 (54.0%) |
| Urinary | 28 (18.7%) |
| Pulmonary (suspected or confirmed) | 5 (3.3%) |
| Intracranial (suspected or confirmed) | 4 (2.7%) |
| No bleeding reported | 15 (10.0%) |
